# Becoming and unbecoming academics: Classed resources and strategies for navigating risky careers

**DOI:** 10.1111/1468-4446.13165

**Published:** 2024-11-14

**Authors:** Marte Mangset, Julia Orupabo

**Affiliations:** ^1^ Department of Sociology and Human Geography University of Oslo Oslo Norway; ^2^ Institute for Social Research Oslo Norway

**Keywords:** academic careers, class, classed resources, disinterestedness, practical consciousness, strategic navigation

## Abstract

Academics influence not only knowledge production but also selection to the labour market and policy development. They have power. Despite the sociological attention paid to class in higher education, few studies have examined the way in which class interferes with the careers of those navigating from being students to becoming scholars. Building on Bourdieu's theory of social reproduction, this study examines how class influences different groups' experiences of becoming academics. Based on 60 interviews with Norwegian scholars in their early to mid‐careers, the analysis identifies the kind of classed resources that are in play in the unequal access to academic positions. Beyond more classical resources, such as financial, cultural, and psychological certainty, the interviewees point to the significance of an early familiarity with the rules of the game and strategic navigation of the academic system. We use these findings to discuss and nuance Pierre Bourdieu's perspectives on the role of incorporated, practical consciousness and disinterestedness in class reproduction in the academic world. This theoretical contribution facilitates the combined analysis of the implicit *and* the explicit ways that dominant classes preserve their position in the hierarchy, which the study demonstrates as key to social reproduction in academic careers.

## 1 INTRODUCTION

Contrary to political expectations, a substantial body of sociological work shows that educational institutions are important agents regarding reproducing class differences (Bourdieu & Passeron, 2000; Goldthorpe, 2000; Reay et al., 2001). Working‐class students are constrained and often opt out of higher education (Archer & Hutchings, 2000; Reay et al., 2009), classed resources influence students' educational progress (Marbach & van Zanten, 2023), and working‐class students deal with ambivalence (Lee & Kramer, 2013; Reay et al., 2009). However, less is known about how different groups navigate from being students to becoming scholars and whether class continues to structure academic careers (Crew, 2021, 2024).

Positions such as that of a professor in a university are key to defining what counts as valuable knowledge in society and informing public debate and political decision‐makers. Through the grading of students, academics also influence selection to the wider labour market. Academics' institutional and market position, their access to several types of resources relative to what other social groups have, and their capacity to influence others' chances of mobility in a stratified society, makes it relevant to use a concept of class in line with Pierre Bourdieu's theories, and to situate academics in a dominant class position (Bourdieu, 1984; Flemmen, 2013).^1^ A key question is thus also how class influences academics' careers and access to such dominant positions as well as to their relative position within the academic field. This paper examines how early career academics consider class to be relevant to their sense of belonging and their chances of success in a competitive and risky academic labour market and it contributes to developing the theoretical understanding of class reproduction in academia.

One reason for privileging research on students over that on academics may be the findings by quantitative scholars that indicate the declining impact of social origin the higher one ascends in the educational system (Mare, 1980; Stoltzenberg, 1994). Although some statistical studies (Mastekaasa, 2006) describe classed differences in students' enrolment in doctoral programmes, which we can consider the first step in an academic career, these differences are interpreted as an indication of students' preferences, not as an indication of variation in the students' resources linked to their social background. Contrary to the idea that social reproduction in academia is primarily linked to preferences, qualitative studies of working‐class students embarking on academic careers show that these, who seem to prefer such careers, still feel less prepared for it than their middle‐class colleagues. By using terms such as ‘strangers’ (Pifer et al., 2023; Ryan & Sackrey, 1995) and ‘space invaders’ (Puwar, 2004), scholars have demonstrated that despite newcomers' upward mobility, they often feel stigmatised (Crew, 2021, 2024) or ‘out of place’ in spaces that have historically been predominantly occupied by the middle‐class (Archer, 2008; Brook & Michell, 2012; Puwar, 2004; Reay, 2004). However, much of this literature draws on sociologists' autobiographical accounts (Brook & Michell, 2012; Pifer et al., 2023; Reay, 2004; Ryan & Sackrey, 1995). Systematic empirical studies of the experience of the role of class in academics' careers are scarce.

Further, recent studies have suggested that class not only influences identity and emotions but also structures academics' ability to position themselves in a hierarchical academic landscape with varying working conditions. That is, working‐class academics often perform academic labour in ways that diverge from the established norms, which are predominantly shaped by middle‐class and elite values, and they end up occupying positions in institutions perceived as less prestigious (Binns, 2019; Rickett & Morris, 2021). However, we do not know enough about how class may matter to selection into different positions in the hierarchy of academic careers. The existing literature, both quantitative and qualitative, calls for further and more systematic empirical investigations into how class is experienced and may interfere with academic careers.

Building on qualitative interviews with 60 early career academics with working‐class and middle‐class backgrounds in Norway, this study analyses how the interviewees experience classed resources and risks in a competitive labour market. Further, we find that our interviewees who experience their academic careers as comfortable and successful describe an explicit, interested, and strategic approach to navigating the academic system. Those who experience their careers as less rewarding argue that they discovered the need for strategic navigation too late and that they have not been able to master the rules of the game. In sum, these findings are, to a large extent, in line with Bourdieu's (1990, 1998, 2000) classical theories of social reproduction, in particular on the role of cultural capital, an academic habitus, and an incorporated practical consciousness, but they also give us reason to discuss and adjust parts of these theories. With this article, we thus contribute with an empirically founded and nuanced theoretical understanding of the role of both tacit, practical consciousness and more explicit, discursive consciousness and strategic navigation in social reproduction in academia.

Below, we will present the Bourdieusian theoretical perspectives on the role of class in social reproduction in academia. Then, we will display some key features of the Norwegian context and the data and methods used in this study. The following three sections will present the analysis of the empirical data, focussing, first, on the classed resources pointed to by the interviewees and, second and third, on the interviewees' descriptions of their more or less strategic navigation of the academic labour market. In the final section, we discuss the implications of the study for theoretical understandings of social reproduction in academia.

### 1.1 Embodying a feel for the academic game

Several of the qualitative studies of classed academic careers mentioned above draw on Bourdieu (1988) and his conceptualisation of the academic field as an arena in which the prevalent types of knowledge, tastes and ways of being are those of the middle‐class and where people from the working‐class struggle to read and capitalise on the ruling norms and culture (Crew, 2021, 2024; Pifer et al., 2023; Reay, 2004). The knowledge and tastes favoured in the system are not favoured because they are inherently better, but because they are the ones that belong to the dominating class and defined as better by them. Key to Bourdieu's understanding of how the middle‐class manages to reproduce their own favoured access to and dominant position in this arena is his practice theory and concept of *habitus*. He posits that individuals carry a collection of embodied techniques and beliefs formed by past experience and socialisation; an embodiment of social position (Bourdieu, 1991; Jawitz, 2009). Thus, ones' habitus is related to the amount and kind of resources one has had access to during early and secondary socialisation, Bourdieu used the terms economic and cultural *capital* to stress the way these resources are convertible into power and privilege. Habitus is not only related to the past, but also a capacity to enter *new* situations and act in a way that makes sense precisely based on our experiences.

The practical sense is what enables one to act as one ‘should’ (*ôs dei*, as Aristotle put it) without positing or executing a Kantian ‘should’, a rule of conduct. The dispositions that it actualizes – ways of being that result from a durable modification of the body through its upbringing – remain unnoticed until they appear in action, and even then, because of the self‐evidence of their necessity and their immediate adaptation to the situation.

(Bourdieu, 2000, p. 139)

Bourdieu argues that these ways of being in the world are so efficient in contributing to reproducing class relationships *because* they are incorporated, tacit and taken for granted. We do not explicitly think about how to act in classed ways, we simply do so:

Practical sense, social necessity turned into nature, converted into motor schemes and body automatisms, is what causes practices, in and through what makes them obscure to the eyes of their producer, to be sensible, that is informed by a common sense. It is because agents never know completely what they are doing that what they do has more sense than they know.

(Bourdieu, 1990: pp. 68–69)

The existing literature on the role of class for students in the educational system and the few existing studies on the classed experiences of academics point precisely to such tacit, embodied ways of behaving, which vary with social background. They show how working‐class students and working‐class academics struggle to act in ways that ‘make sense’ in the middle‐class environment that academic institutions represent. Bourdieu himself argues that people from the middle‐class, and people with the right cultural capital, possess a habitus that enables a ‘feel for the game’, an unconscious, practical mastery of the implicit rules of success (Bourdieu & Wacquant, 1992). Having a ‘feel for the game is having the game under the skin’ (Bourdieu, 1998, p. 80). Assessing the practices of succeeding in academia involves tacit knowledge that cannot be described, because the ‘assessor's knowledge exists in the practice of the skills and not in a set of published maxims’ (Jawitz, 2009). The question is whether we, in the empirical study that we have conducted among junior academics in Norway, find such differentiating experiences of a tacit ‘feel for the game’ on one hand, and a lack of ‘feel for the game’ and struggles to fit into the academic environment and a ‘habitus clivé’ (Bourdieu, 2004; Friedman, 2016) on the other.

One of the characteristics of the academic field as conceptualised by Bourdieu is a logic of *disinterest*, that is, that the actors in the field should not express an explicit interest in ‘winning the game’:

If disinterestedness is sociologically possible, it can be so only through the encounter between habitus predisposed to disinterestedness and the universes in which disinterestedness is rewarded. Among these universes, the most typical are, along with the family and the whole economy of domestic exchanges, the different fields of cultural production, the literary field, the artistic field, the scientific field, and so forth, microcosms which are constituted on the basis of an inversion of the fundamental law of the economic world and in which the law of economic interest is suspended.

(Bourdieu, 1998, p. 88)

Rather, in this field, by acting disinterested, one may ‘win the game’, although as a ‘biproduct’ or unintended consequence. According to Bourdieu, winning the game should not be an explicit strategy; that would make it less effective:

…the most effective strategies, especially in fields dominated by values of disinterestedness, are those which, being the product of dispositions shaped by the immanent necessity of the field, tend to adjust themselves spontaneously to that necessity, without express intention or calculation.

(Bourdieu, 2000, p. 138)

Thus, this perspective suggests, first, that academics who succeed do so because they have an embodied ‘feel for the academic game’, not because of conscious calculation or strategic action and, second, that those who have a feel for the game need not craft a plan or objective for their practice, because they are absorbed in doing (Noble & Watkins, 2003). Practical and embodied knowledge, emanating from an upbringing in a home with the right amount and type of capital, is what allows the middle‐class to succeed with ease; it is not something one strives for; it comes ‘naturally’. As Bourdieu states, ‘One can have a magnificent academic career without ever needing to give oneself such an objective’ (Bourdieu, 1998, p. 82).

According to Bourdieu, the working‐class can become aware of what the game is about and attempt to learn it, but because they do so in a conscious and ‘scholastic’ way instead of automatically and with ease, they will be less efficient than others. Conscious and reflexive engagement in practices only occurs when there is a lack of fit between habitus and field (Adkins, 2004; Bourdieu & Wacquant, 1992). Reflexivity is understood as an ‘awakening of consciousness’ and a state of ambivalence that characterise unstable positions, such as when people experience upward or downward mobility (Bourdieu, 2000; Bourdieu et al., 1999). Based on the many studies showing Bourdieu's theories' pertinence in higher education, as well as the aforementioned studies of working‐class academics' sense of awkwardness, we would expect to find similarly classed practices of embodied ease and disinterestedness among middle‐class academics, as opposed to more conscious, learnt and uneasy practices among working‐class academics.

## 2 THE STUDY

### 2.1 The Norwegian context

Norway is generally perceived as a country with small social and economic differences due to its small size (five million inhabitants), prosperous economy, social‐democratic political tradition, and lack of nobility (Bendixsen, Bringslid, & Vike, 2018). Nevertheless, recent studies have indicated significant economic inequality in Norway, particularly concerning wealth (Hansen & Toft, 2021). Even in this egalitarian country, class has been shown to matter in politics (Flemmen & Haakestad, 2018), culture (Flemmen et al., 2018) and school grades (Hansen & Strømme, 2021; Strømme & Wiborg, 2024). Despite these findings, Norwegians tend to perceive themselves as a population not marked by class hierarchies (Hjellbrekke & Korsnes, 2012). Thus, it is pertinent to speak of a ‘silence of class’ (Kolehmainen, 2017) in Norway. However, regarding academic careers, where scholars rather have focused on persisting gender inequalities (Orupabo & Mangset, 2021), studies of the role of class are scarce, as they are in other European countries.

Although the Norwegian higher education system is clearly less hierarchical than those in other European countries, there is still a hierarchy of institutions, of departments and of positions. Most higher education institutions have been granted university status, but with varying levels of prestige between universities providing vocational training and universities offering training in classical academic disciplines (Bleiklie, 2023). There is also a significant labour market for academics in applied research institutes. The extent to which one can spend time on research, as opposed to teaching, and the degree to which one can define for oneself what kind of research one can conduct, are relevant to the notion of hierarchy. The issue for our interviewed academics is not simply whether they can obtain a position in the academic field, but in which part of academia and under which working conditions they can do so.

### 2.2 Methods and data

The analyses in this article are based on data gathered as part of a larger study of recruitment to academic positions and academic careers. For this article, we make use of 60 semi‐structured interviews with early and mid‐career academics in the disciplines of history, political science, and biology. The interviews generally lasted between 1 hour and 90 min. Most of the interviewees held temporary positions, such as postdocs and researchers at universities. A small minority had recently obtained permanent positions as associate professors or professors at universities. Some held permanent positions as researchers in applied research centres. The interviewees were recruited from higher education and research institutions across the country. Using staff information from the institutions' websites, we constructed a list of potential interviewees from those who were postdocs or had completed their PhDs within the last 7 years. We contacted most of these and interviewed those who answered. Almost all those who were contacted answered.

The interview guide contained questions on the interviewees' paths into an academic career, their successes and losses and their perceptions of the labour market, their perceptions of selection criteria, and their positions in the market. We began by asking open, descriptive questions to build rapport and avoid introducing pre‐defined analytical frameworks (Spradley, 1979). Later in the interviews, we introduced questions on more specific topics. Whereas how the interviewees perceived and navigated the academic labour market was part of our initial interest, the issue of class was not. However, family background – sometimes including the concept of class ‐ and what it had meant for choosing and handling entry into an academic career arose at the interviewees' initiative in a large part of the interviews. Therefore, we decided to follow up on this issue in our analyses.

As the study took place during the COVID‐19 pandemic, most of the interviews were conducted on Zoom, with only a few (7) being carried out live in the interviewees' offices. The interviews were conducted by either one of the two authors or a research assistant in Norwegian (except for five interviews done in English at the interviewees' request) and later transcribed verbatim. Our own situation, as early to mid‐career academics, was likely central in building rapport and facilitating extensive answers (Spradley, 1979). We coded the interviews in NVivo with a combination of predefined codes, such as perceptions of the labour market and of criteria for success, and codes constructed based on new findings in the interviews, such as classed experiences. Each author coded the interviews conducted by the other. The quotes from interviews conducted in Norwegian are translated by the authors.

The sample is almost gender balanced (32 women, 28 men). Out of the 60 interviewees, 46 (24 women, 22 men) had parents who had completed higher education, 13 (seven women, six men) had parents with little or no higher education and one (man) interviewee's background is unknown to us. As class initially was not part of our analytical framework and only emerged as a central theme during the interviews and analyses, we had not gathered extensive information about the interviewees' class background. We systematically asked for parents' level of education, however, not for their occupation, financial situation or other dimensions of class which would have given a broader picture relevant to an analysis of classed resources in academic careers. However, as the interviewees' introduced the role of their family background and various types of resources related to that, and often also the class concept, during the interview as relevant to making sense of their academic trajectory, we considered that this justified using the class concept in our analysis. The interviewees' own identification with working‐class or middle‐class background, as well as their referral to their parents' level of education or academic background, was decisive to how we categorised them in our study. Further, the sample and data are not suited for generalising about working‐class academics' chances of success in the academic system. Hopefully, statistical data and more detailed information about class background among academics in future studies, may shed more light on the role of different dimensions of class background to academic careers. In this study, rather, the rich interview data and self‐perceptions of class background offer insights into early career academics' understandings of the academic labour market, ways of navigating it, and how entry into the academic career system may be experienced as classed and contribute to reproducing classed relations in academia.

## 3 FACING RISK WITH CLASSED RESOURCES

Overall, our interviewees described entering the academic labour market as a hazardous endeavour and stated that the risk of waiting a long time for a permanent position, experiencing periods of unemployment, not obtaining an academic position at all, or not obtaining one that allows for the kind of research activity they aspire to, was real. The interviewees, across disciplines, genders, and social backgrounds, pointed to the strong competition in this labour market. However, in facing this competition, they do not all have the same resources. Although there is no clear‐cut connection between class background and career success in our data, the interviewees bring up varying resources—or the lack of such—which it is meaningful to describe as classed.

The informants with a working‐class background describe constraints in relation to economic uncertainty, a lack of social support and subtle cultural exclusion in their pathways to becoming academics. Having the capacity to wait for a job opening and remain in temporary jobs despite uncertain prospects was interpreted as necessary to succeed in obtaining a permanent position. Economic resources in the family were perceived as relevant to how this uncertainty was experienced. One of the interviewees with working‐class background noted the following:

Since I don’t come from a particularly rich family, I had to move very far from work (due to housing costs near the university), which also influences my capacities. (…) many of my colleagues have very privileged backgrounds … They have expensive apartments on the west side of the city. We don’t talk much about that. We talk more about the gender dimension. (…) If it continues like this, they will favour those who have privileged backgrounds, and academia will be a game for … well, probably not the regular guy (…) To wait [in] temporary positions for so long, that it is a problem for a mortgage and that sort of thing.

(Man, historian, working‐class)

Compared to his colleagues who come from more privileged backgrounds and live near the university, he has shorter working days due to his commute. In this way, a lack of economic resources, of economic capital in Bourdieu's terms, influences his capacity for career devotion. We also see that the informant experiences that in contrast to gender issues, this type of class‐based differences is not recognised or ‘talked about’ in his institution. The ‘silence of class’ in egalitarian Norwegian society is something several of the informants with a working‐class background mention as a problem.

Becoming an academic is also experienced as riskier for the informants with a working‐class background due to their lack of social support from family and friends, which may be related to classed access to information about mobility opportunities and career paths. The working‐class academics noted how their lack of familiarity with academia made it difficult to imagine career possibilities in academia as young students. The historian quoted below felt that being the first in her family to enter the university, the decision to choose an academic career was difficult. Where she hails from, it is not considered a proper job:

So, I’m from a family where I’m the first to ever enter university. And for me, I was always interested in history. For my family, this was not a proper job, so everyone wanted me to do a traineeship in a bank or something like that—a proper job. It was a bit hard to really decide and say … ‘No, I am going to university to study history’, which is not something like [becoming an] engineer, [a] lawyer or … a physician, of course. (….) So, it was really just being curious about these topics and then making my way, without any real idea about what a career path might be afterwards. (…). I was, at that point, studying for a master’s degree [in history] and taking teaching education because becoming a teacher is at least something your family considers … like a job.

(Woman, historian, working‐class)

In contrast to those on a purely scholarly career track, this interviewee had also invested time in an alternative and less risky route. Thus, a lack of support and informal knowledge about academic careers makes the transition from student to scholar more time consuming. Scholars with different class backgrounds possess quite different resources in dealing with self‐doubt and hardships while navigating from being students to becoming scholars. This self‐doubt should also be considered in relation to the cultural and economic capital she draws on and how these two types of resources are intertwined: her family does not have the information on what an academic career path is like, and they don't have the economic capital to take lightly that their daughter embarks on a career that might not procure a secure income.

The risk associated with pursuing an academic career is also related to the informants' *sense of place* in academia. While the informants with middle‐class backgrounds to a larger extent see their place in academia as natural, the working‐class academics experience emotional and psychological uncertainty. Interviewees from the middle‐class highlighted how coming from the right kind of background may have enabled their career progress through feelings of belonging.

I had a Bourdieu moment when I started my master’s degree in political science and suddenly understood very well the difference between coming from an academic background and not coming from one. It is obvious that I have brought with me a kind of confidence about belonging [in academia] that many others don’t have. I have good friends and colleagues who have expressed that it is some kind of … not arrogance but a feeling of self‐evidence that obviously has been of help. It is not necessarily a demographic background variable but a cultural background variable—you can call it that—which I have used Bourdieu to reflect about. Knowing the codes, being comfortable in an academic environment, taking it as a given that you belong—it is obvious that that has mattered.

(Man, political scientist, middle‐class)

This interviewee hails from a family of professors, including his parents, grandparents, and great‐grandparents. From his early student days his surroundings considered his presence in academia natural. By positioning himself as part of the academic middle‐class, the interviewee interpreted his sense of place and confidence in academia as classed expectations and resources. The cultural capital that his family passed on to him, and the habitus he developed, helped his feeling of belonging in the academic world.

In contrast, the first‐generation academics described academia as an unfamiliar field and perceived a lack of belonging, in line with the above‐mentioned literature on working‐class academics feeling out of place (Crew, 2021, 2024; Pifer et al., 2023). An interviewee with a working‐class background described how his unfamiliarity with academia and experiences of exclusion materialised through subtle distinctions:

It is very subtle things. So, it’s not that I must acquire a specific culture, but it is kind of about ‘Where do you live?’, ‘What do your parents do?’, ‘Where are you going on holiday?’ [and] ‘What music do you like?’ It’s things that you’ve had from childhood. It’s very subtle, so it’s difficult to point to anything specific. It’s more … it’s the feeling of knowing the right people or not (…) the language, what you laugh about, the sum of tiny, subtle details. And I find it very strange that this is not an issue in academia when one, and rightly so, discusses gender discrimination and, maybe to a lesser degree, racism, as there are hardly any foreigners in Norway. But this cultural and social discrimination, that is never discussed. Many of my colleagues are sons of academics, and I can see that, somehow, they have more success in their careers.

(Man, historian, working‐class)

By pointing to what he refers to as the sum of small, subtle details, the interviewee describes a feeling of being out of place. His own cultural resources do not fit with the type of culture dominating academia; he lacks the cultural capital fitting with the academic field. Although he has obtained a permanent position, he interprets what he considers constrained possibilities for career progression as related to his background. He immigrated to Norway from a Western country and has experienced that the Norwegian egalitarian context makes it especially difficult to address barriers related to class. He felt that in contrast to in his home country, the language used to address class is absent in Norway. Although we have not conducted a comparative analysis, people's responses to exclusion may be enabled or constrained by the cultural repertoires available in various countries (Lamont et al., 2016). As previously shown, informants with Norwegian backgrounds also problematise the silence of class when making sense of their experiences as working‐class academics.

While the possession of different types of classed resources enables the middle‐class to become academics through feelings of fitting in, those with working‐class backgrounds experience constraints and uncertainty because they lack these resources. The interviewees point to aspects that can be defined in terms of economic capital and in particular they point to aspects that can be defined as cultural capital as relevant to their feeling of fitting in. However, beyond these classical classed resources, Bourdieusian perspectives on class reproduction also stress the importance of the feel for the game. Below, we will explore whether and how the mastery of the rules of the game come into play according to our interviewees.

## 4 STRATEGICALLY NAVIGATING THE ACADEMIC LANDSCAPE

Regardless of class, our interviewees described the academic labour market as fiercely competitive. How did they navigate this competitive landscape? How did class interfere with this?

The interviewees, across classes, had a clear picture of what counts in the competition for jobs. Although several criteria were mentioned, including having won research grants and connections to prestigious international institutions, the most highlighted criterion was publications. The interviewees described the successful academic, the one that obtains a permanent university position within a reasonable time, as an efficient scholar with an impressive publication record. What they also seem to agree on is that to have such a track record, one must distinguish between promotable tasks and those that are obstacles to a successful career. Thus, interviewees both from middle‐ and working‐class backgrounds spoke explicitly about the rules of the game and the need to play by them to succeed.

My experience is that it is very much about the quantifiable parts of academia. So, having experience with teaching counts for little. That’s my advice to PhD students now: do not be nice and take on more teaching obligations. What I mean is, do exactly as much as is required of you, but do not believe that taking on extra teaching or extra grading will have a positive impact. Be cynical and know that what counts is publishing.

(Woman, political science, middle‐class)

Most of the interviewees take pleasure and find meaning in several parts of academic jobs; they stated they enjoy both teaching and research. However, they acknowledge that these tasks are differently positioned in an academic hierarchy of worth. The question is thus whether one manages to navigate in this system to find a permanent position in the right institution and the right department. Our interviewees had varying experiences in this regard.

Interviewees with middle‐class backgrounds described how they strategically manoeuvred regarding accumulating tasks they perceive as crucial to their academic careers. The most obvious adjustment to be made is generally the type and number of publications. They indicated that they carefully navigate to carve out time to write the right kind of publications and position themselves in their fields. This manoeuvring could also be a question of choosing the right method and topic to increase one's chances in obtaining a position. The interviewee in the quote below is from a middle‐class background. To succeed in becoming an academic, she decided to reframe her research topic, which was education policy, in such a way that it was perceived as relevant to a larger part of the discipline:

… if we focus only on [the research topic] education, we’re going to have a hard time finding the next position, so we should start expanding our profile – which doesn’t necessarily mean … [for] me, shifting entirely to another policy domain. It meant more that I changed or was gradually changing where I was submitting my articles. So, these were no longer journals specialising in education, but more mainstream journals on policy analysis.

(Woman, political scientist, middle‐class)

First, the interviewee recognised that research on education is not as prestigious as other topics within political science. Second, she realised that if she sent her articles to a different kind of journal than she had initially and changed the framing and literature discussed, she could make her research and publication profile more attractive to future academic employers. To do something like this, one must be well informed about the functioning of the system. The strategy paid off for this interviewee, who succeeded in obtaining a permanent university position.

Another aspect several of our interviewees mentioned is how different methodological approaches are valued differently in the academic system. They spoke of their degree of mastery of the preferred methods, how they had taken that into account or not in their work, and the relevance of this to their level of success. The political science interviewees generally perceive mastering quantitative methods as an asset for an academic career. Their awareness of this and capacity to act on it matters:

I work a lot with quantitative methods, and I got an impression… that is perhaps also the reason why I work with quantitative method myself … because I got the impression that it is easier to get these types of studies published in the best journals in political science. So, I think that, probably, has something to say [about succeeding in getting a good position]. I think it is more difficult to get things into the top journals if, for example, you work exclusively qualitatively…

(Woman, political science, middle‐class)

The capacity for strategic navigation may be even more significant for those who do not initially have the most highly valued competence:

B There is method discrimination, so if you have a qualitative—only qualitative—background today, that is not so good. You should have quantitative skills. (…) So, I am, in many ways, not quite set up for today's world. But that's how it is.

A You don't work quantitatively at all?

B No, I'm trying to learn some skills.

A It's something you think you need?

B Yes. It is about what the Research Council values, but I also think the journals are increasingly pulled in that direction. Journals with high impact want quantitative articles. I try to solve it by working with people who have that experience and by co‐authoring. Then, you can add what you are good at. [But I also try to learn new skills.] I run and create datasets during the day, so it's probably going in the right direction. (Man, political science, middle‐class)

The interviewees thus point to both the need to understand what the system values and the rules of the game and the need to adjust one's own research and competence profile to adapt to these rules. The middle‐class academics quoted above managed that.

## 5 THE WORKING‐CLASS'S LATE DISCOVERY OF THE RULES OF THE GAME

Other interviewees interpreted their chances of obtaining the positions they once dreamt of as unlikely due to their late discovery of the rules of the game, that is, to the fact that they had spent their time and energy on a type of academic labour that was less appreciated in the system and which counted less for hiring and promotions, such as teaching, taking part in hiring committees or other types of ‘academic housework’. Spending more time abroad was also highlighted as something they hadn't prioritised or had the opportunity to do, but also that they had failed to realise its importance at an early stage of the career. The working‐class academic in the quote below points to how the tasks she had prioritised thus far hindered her from obtaining a university position and the fact that it was difficult to imagine how she could position herself better now:

A So, you think that in that kind of competition, you would never succeed, and you don't even think of that kind of [university] position as relevant?

B No, I would have had to start over again—turn time back and be more goal oriented.

A What would you have done differently?

B At least spent more time abroad and been more focused on publishing than anything else.

A What have your priorities been?

B Well, we've had a lot of students, and since the group has been very small for a time, and the professor was very busy with other things, I was the only one [who could deal with the daily tasks] (…) For a long time, I was not very productive [publishing wise]. Maybe changing the environment after the PhD would have done me some good; that would maybe have been a good idea, but I didn't know that then. (Woman, biologist, working‐class)

The working‐class interviewee quoted below states that it took him a long time to realise what parts of academic labour lead to opportunities:

I didn’t know academia, really. I come from a working‐class background, and for my parents, the university is a completely foreign universe. And it has also always been that to me, right? So, how things work, as well as what it takes and all that, that has been completely unknown territory for me (…) It is something that most others, [this] is my impression, know very well before they even start (…) The consequence is that I have had to, in addition to working here, had to figure out the system or figure out the academic culture, what this is (…).

If I had known all I know now before I started here, I think I would have gotten further in my career (…) I would have been closer to professor level. I would simply have done more research (…) All these things that many others have been doing for years before they finally get a job, I have … it has been completely unknown territory for me. All those things that are assumed to be known, I haven’t known anything about them. What are my options, sort of? …What do I do to apply for grants, to get more time for research? What are the possibilities? There are loads of possibilities, but I didn’t know about them.

(Man, historian, working‐class)

Coming from a working‐class background, the interviewee did not know how to navigate to succeed. It took him a long time to realise that there are ways to devote more time to research and have better work conditions, which again increases the chances for hiring and promotion. The interviewee noted a lack of information as the reason for his slower career progress as compared to others. If he had possessed strategic knowledge at an earlier stage of his career, he would be closer to obtaining a professorship.

The working‐class academics, lacking the relevant cultural capital and who discover the rules of the academic game too late and fail to master them, can certainly be considered to conform to Bourdieusian theory regarding how those who move up in the class hierarchy end up being conscious of but not mastering the game. However, our data suggest that these working‐class academics are not those *most* conscious of the rules of the game.

## 6 DISCUSSION AND CONCLUSION: NUANCING THE LINKAGES BETWEEN IMPLICITNESS AND POWER

Despite the sociological attention paid to class in the literature on education, only a few studies have examined the experiences of those who navigate from being students to becoming scholars and how class may influence their academic careers (Crew, 2021, 2024). As top positions in the academic system offer both power and privilege, there is a need to outline the paths into hierarchised academic positions and how these may be classed. Based on analyses of 60 interviews with early and mid‐career academics in Norway, we found that the experience of entering academia is classed in three ways.

First, our interviewees pointed to classed resources that were relevant to their career success: economic resources, cultural resources, and social support. The resources they pointed to can fruitfully be described with Bourdieu's concepts of economic and cultural capital. While economic capital in the family helps attenuate the economic risks related to a career in a competitive academic world, cultural capital helps deciphering what an academic career is and how to fit in in an academic environment. Among our interviewees, there were clearly different levels of resources to draw on between those coming from a working‐class background and those coming from a middle‐class background, here defined as having parents with higher education. However, the ones having family members that were academics appeared as particularly well endowed with useful cultural capital.

Second, the interviewees spoke of the need to know and master the rules of the academic game, and some of them pointed to how social origin mattered regarding whether they were familiar with these rules or not. In particular, the interviewees pointed to the need to be familiar with these rules *in time* to orient one's academic work in the right direction for career success. The ones among our interviewees who were the first in their family to go to university, and which we categorised as working‐class, argued that their late discovery of the rules of the game had hindered their academic career progression. Their lack of familiarity with the academic world, their lack of relevant cultural capital, had consequences for their careers.

Both these factors, classed resources and timely familiarity with the rules of the game, are in line with Bourdieu's theories of social reproduction. The way the interviewees point to subtle distinctions relevant to the feeling of belonging or being out of place seems quite in line with Bourdieu's understanding of how certain types of knowledge and ways of being are embodied and not primarily operating at a discursive, reflexive level.

However, there is a third factor that the interviews unveil as relevant to career success: a very explicit and consciously strategic way of navigating the academic labour market. The middle‐class academics interviewed in this study, and the ones coming from families of academics, demonstrate something which is different from an implicit and automatised way of acting in this field; they are far from the academics who lack the objective of an academic career, as Bourdieu described. Rather, they have explicitly and clearly prioritised work tasks that pay off in the academic system. They explicitly state that they have acquired new methodological skills, entered co‐authorships, and adjusted the framing of their research with the explicit ambition of improving their career chances. They do not act in a disinterested way, and that seems to help them win the game, that is, obtain the best positions in the academic hierarchy.

This is not in line with Bourdieu's theory on how an embodied, implicit, and disinterested feel for the game is the most efficient way of acting in the academic field. Our point is not that such an embodied, automatised feel for the game is not helping the middle‐class and those with most cultural capital and an inherited academic habitus. There are surely indications that this is also an important part of the mechanisms of social reproduction. However, we interpret the latter part of our findings as an indication that we *also* must pay attention to explicit, conscious, and interested strategies if we are to understand social reproduction in contemporary academic careers.

How should we interpret this difference between the way social reproduction takes place in contemporary Norwegian academic careers and what Bourdieu and others have described in academic systems in other countries and other times? One possibility is that reproduction in Norwegian academia is somewhat different from, for example, that in France and always has been due to the specificities of the Norwegian academic system. Weininger and Lareau (2003) show how some mechanisms of social reproduction seem more explicit in American education than what Bourdieu found in French education. Another possibility is that the mechanisms of social reproduction in Norwegian academia were more like what Bourdieu describes 40 years ago, but they have changed due to changes in how academic careers are structured. Because we only have data from contemporary Norwegian academics, we cannot determine this here. However, we hope future studies will test whether such a theoretical understanding of social reproduction, including conscious and interested strategic action, on data derived from several countries today so that we can better understand how classed hierarchies are preserved in academic institutions. We also hope new studies which take a class approach from the very start can gather data on more dimensions of class, and include larger samples of academics, to investigate how different aspects of class background may be relevant. In particular, our own findings call for a more in‐depth investigation of the role of class versus the role of coming from a family of academics more specifically.

This study contributes with a systematic analysis of how class interferes with academic careers and adds nuance to Bourdieu's classical theories of social reproduction in the academic field. Our point is not that Bourdieu's theories on the embodied implicit mechanisms of social reproduction were wrong but that we need theoretical tools to understand *both* the implicit and the explicit ways that dominant classes preserve their positions in the hierarchy. The explicit strategies—generally presented and perceived as playing by a meritocratic logic—should not be underestimated as playing a minor part in these processes of reproduction.

## CONFLICT OF INTEREST STATEMENT

We have no conflict of interest related to this manuscript.

## DATA PROTECTION AND RESEARCH INTEGRITY

The project at the basis for the present article is regulated and overseen by SIKT, the Norwegian Agency for Shared Services in Education and Research, to ensure that it is conducted in line with relevant data protection legislation and other research integrity legislation and guidelines. The data gathered for this study is not shared due to ethical considerations regarding the interviewees.

## Data Availability

Data sharing is not applicable to this article as no new data were created or analyzed in this study.

## ENDNOTE

^1^
In line with Bourdieu, we consider academics as dominated dominants in a wider class scheme, based on their relationship with a more powerful economic sector. However, that relationship extends the topic of this article and will not be further discussed here. As we will however discuss further in this article, academics will have different degrees of power within their own field, the academic field. We will examine the role of class to that.
